# Kidney and Inferior Vena Cava Abnormalities With Leg Thrombosis (KILT) Syndrome in a 24-Year-Old Man

**DOI:** 10.7759/cureus.20568

**Published:** 2021-12-21

**Authors:** Mohammad Klaib, Tamara H Alsmadi, Mohammad Momani, Tarek H Alsmadi

**Affiliations:** 1 Internal Medicine, Royal Medical Services, Princess Haya Military Hospital, Ajloun, JOR; 2 Medicine, Royal Medical Services, Princess Haya Military Hospital, Ajloun, JOR; 3 Radiology, Royal Medical Services, Princess Haya Military Hospital, Ajloun, JOR; 4 General Medicine, University Hospital Southampton NHS Foundation Trust, Southampton, GBR

**Keywords:** kidney anomalies, thrombophilia, anticoagulant, ct scan, dvt, ivc anomalies, kilt syndrome

## Abstract

Here, we present a case of kidney and inferior vena cava abnormalities with leg thrombosis (KILT) syndrome, which consists of the triad of congenital kidney anomalies, inferior vena cava anomalies, and deep venous thrombosis. KILT syndrome is usually an incidental finding while investigating other conditions.

## Introduction

The triad of inferior vena cava (IVC) anomalies, kidney anomalies, and venous thrombosis is a rare phenomenon, which was described by Van Veen et al. in 2002 as kidney and IVC abnormalities with leg thrombosis (KILT) syndrome [1], In this case report, we present the case of a 24-year-old male patient with no significant medical history who presented with unprovoked extensive bilateral deep venous thrombosis (DVT), associated with kidney and IVC anomalies. This is the first reported case of KILT syndrome from Jordan.

## Case presentation

A 24-year-old male patient presented to the emergency department with a one-week history of bilateral lower limb pain and progressive thigh swelling. He denied any history of recent trauma or bites and did not report any respiratory or gastrointestinal symptoms. There were no personal or familial risk factors for venous thrombosis, apart from the patient’s eight-pack-year smoking history.

Upon examination, he was conscious, alert, and oriented to place, time, and situation. His blood pressure was 120/70 mmHg, heart rate was 77 beats per minute, respiratory rate was 13 breaths per minute, oxygen saturation on room air was 97%, and temperature was 36.5°C. On examination of the lower limbs, both limbs were edematous, warm, and tender to palpate with prominent palpable cord-like varicose veins bilaterally. Peripheral pulses were intact bilaterally without any signs of limb ischemia. The rest of his physical examination was unremarkable. His initial workup including full blood count, liver function test, and kidney function test was normal.

**Figure 1:**
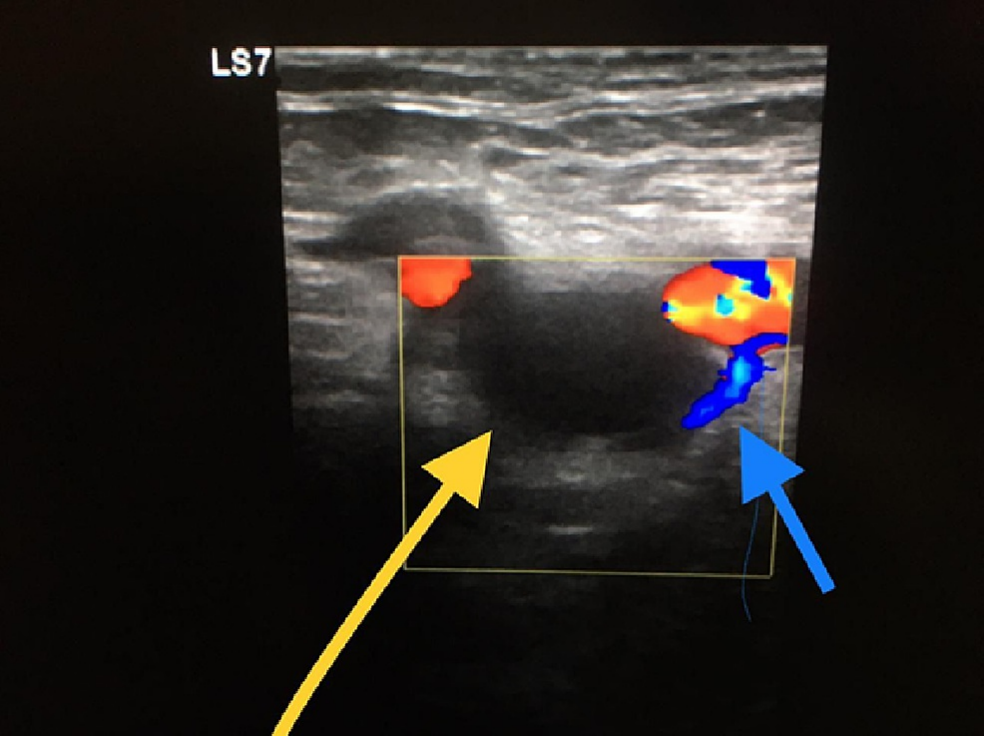
Doppler ultrasound showing empty venous flow (yellow arrow) and partial recanalization (blue arrow).

Doppler ultrasound confirmed the diagnosis of extensive bilateral acute on top of chronic DVT involving the bilateral popliteal, femoral, and iliac veins extending up to the bifurcation of the IVC. There was also evidence of echogenic thrombi with no compressible deep veins and areas of recanalization (Figure 1).

**Figure 2:**
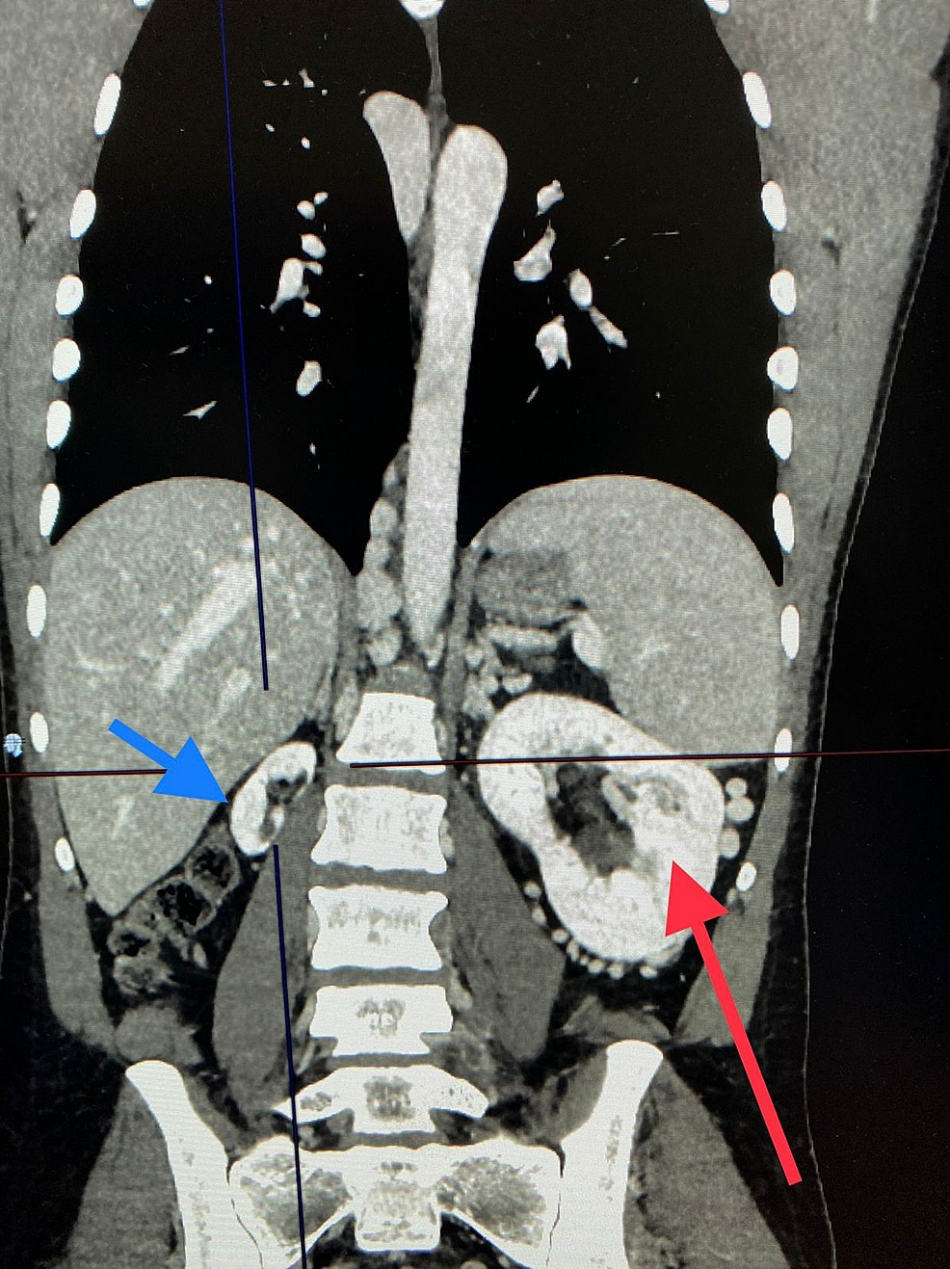
CT scan (coronal view) showing atrophied non-scarred right kidney, suggestive of hypoplasia (blue arrow), and hypertrophied left kidney with moderate hydronephrotic changes (red arrow). CT: computed tomography

A computed tomography (CT) scan of the chest, abdomen, and pelvis was obtained to exclude malignancy as a possible etiology of this unusual presentation. CT did not show any evidence of malignancy; however, incidental findings of collapsed distal IVC with no contrast media filling, multiple collateral venous drainage throughout the abdomen, hypoplasia of the right kidney, and hypertrophy of the left kidney (13 cm in long axis) with moderate hydronephrotic changes not causing an obstruction were noted. The proximal IVC appeared normal (Figure 2).

**Figure 3:**
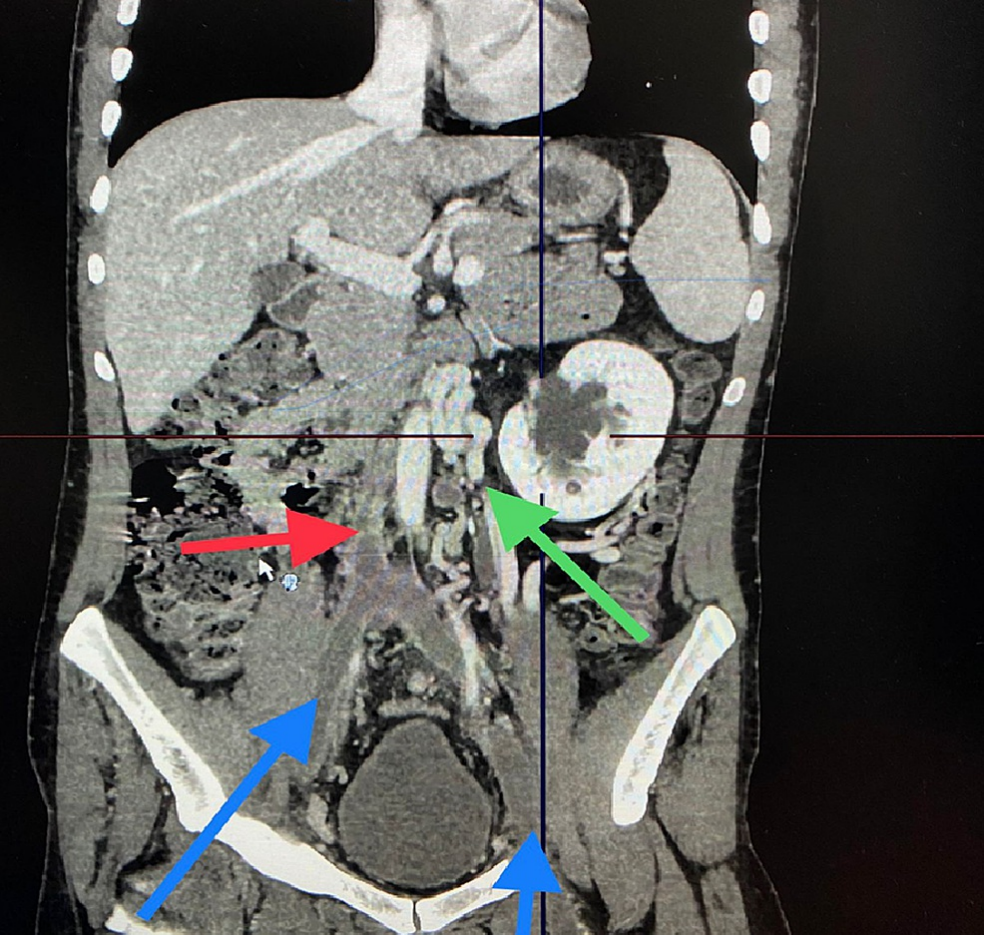
CT scan (coronal view) demonstrating bilateral DVT of the common iliac bilaterally (blue arrows), interruption of the infrahepatic IVC (red arrow), and collaterally draining left kidney (green arrow). CT: computed tomography; DVT: deep venous thrombosis; IVC: inferior vena cava

**Figure 4:**
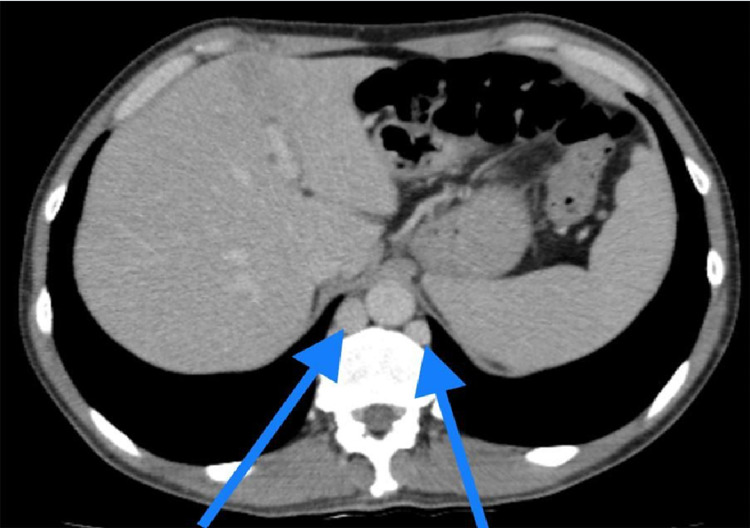
CT scan (transverse view) showing prominent azygous system draining the collateral (blue arrow). CT: computed tomography

**Figure 5:**
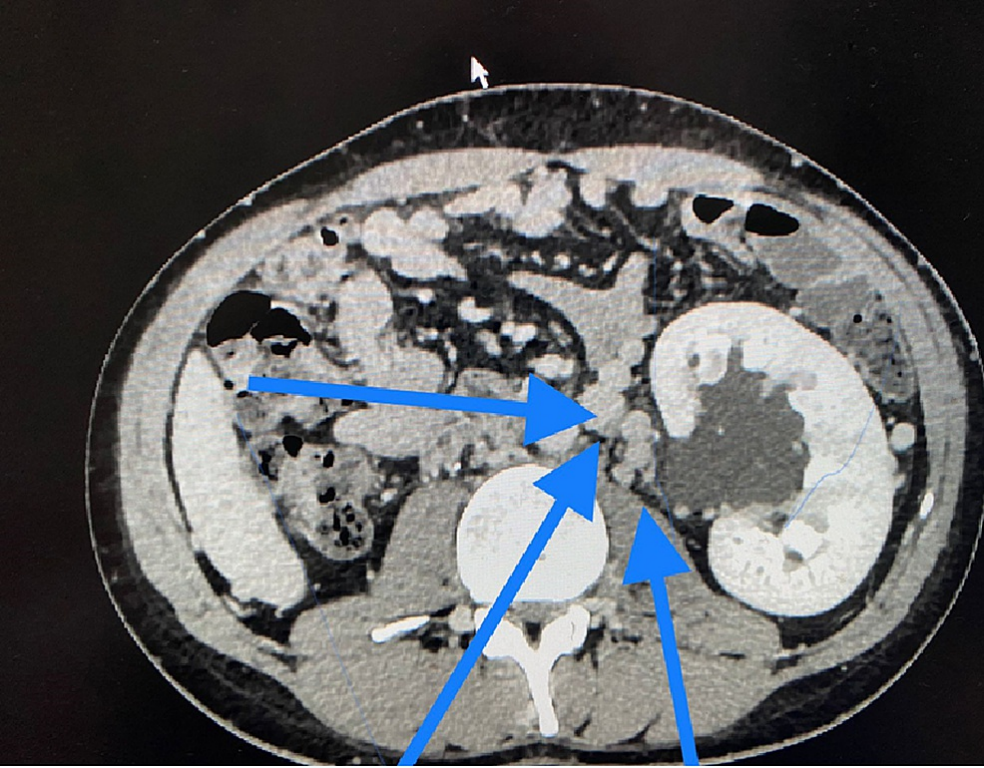
CT scan (transverse view) showing multiple deep collaterals (blue arrows). CT: computed tomography

**Figure 6:**
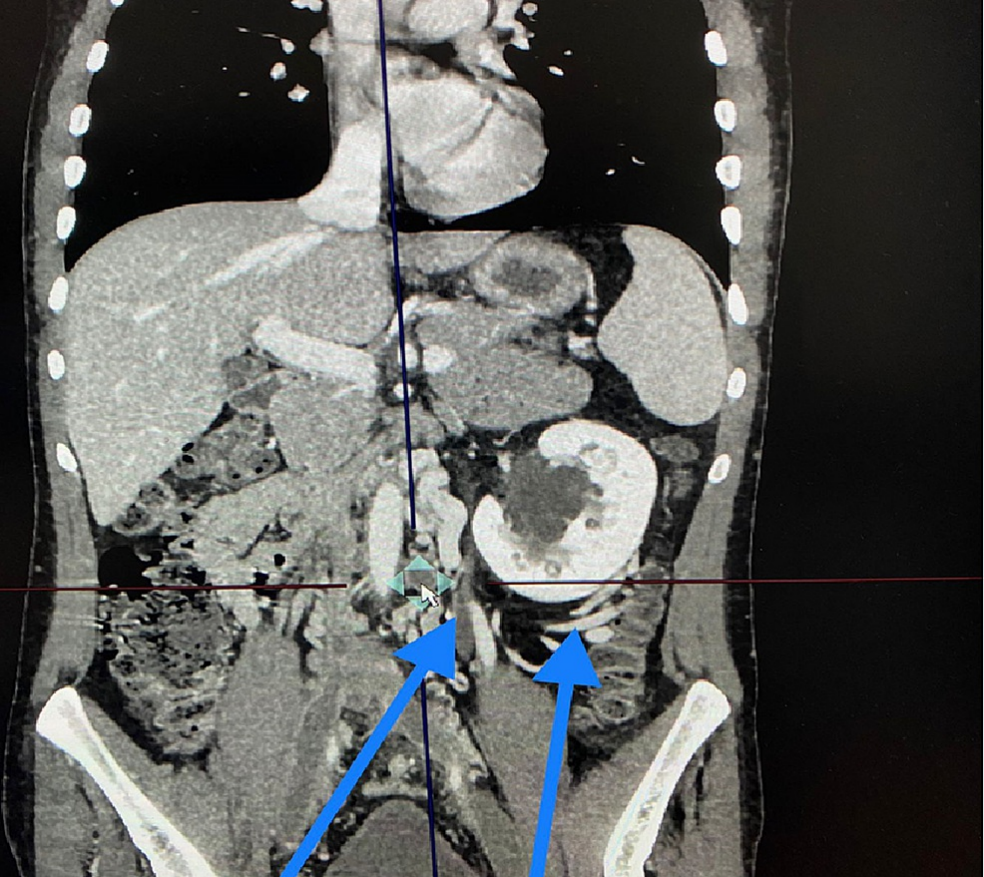
CT scan (coronal view) showing multiple collaterals (blue arrows). CT: computed tomography

A delayed venous-phase CT scan demonstrated IVC interruption (Figure 3) with multiple collaterals draining into the azygous system (Figures 4-6).

Table 1 presents the findings of the thrombophilia workup.

**Table 1:** Findings of the thrombophilia workup. MTHFR: methylenetetrahydrofolate reductase

Thrombophilia workup	Findings
Factor V Leiden mutation	Heterozygous
Prothrombin G20210A	Negative
MTHFR C677T mutation	Heterozygous

Protein C, protein S, and anti-thrombin III serum levels were not tested due to the inaccurate interpretation of their levels during the acute state of DVT as well as the treatment with anticoagulants. The patient was subsequently started on anticoagulation treatment with warfarin and low-molecular-weight heparin to achieve an international normalized ratio of 2-3. Eventually, his lower limb edema and pain subsided with treatment. Upon discharge, he was doing well, was pain-free, and his INR was in the therapeutic range. He was discharged on warfarin. No recurrence of DVTs was reported after 16 months of follow-up.

## Discussion

KILT syndrome, first reported by Van Veen et al. in 2002 [1], consists of the triad of leg thrombosis, IVC abnormalities, and kidney abnormalities. On a literature review, few cases were found.

Agenesis and the absence of IVC can be explained by abnormalities during embryogenesis, which can lead to impaired venous drainage from the right metanephros, resulting in growth abnormalities in the right kidney. The left kidney is not affected due to the alternate pathway of venous drainage; the left metanephros drains via the gonadal vein and lumbar perforators [2,3].

In IVC agenesis, the body compensates for the absence of IVC by extensive collateral formation; therefore, venous flow from the lower extremities becomes slow, leading to increased venous pressure, venous stasis, and extensive DVT formation [4].

The incidence of IVC agenesis ranges from 0.0005% to 1.0% in the general population [5]. During the sixth and eighth week of gestation, IVC develops through a complex process involving three pairs of primitive veins, namely, postcardinal, subcardinal, and supracardinal veins, that appear subsequently and give rise to four segments of adult IVC (hepatic, suprarenal, renal, and infrarenal) [6].

The absence of IVC can be explained by the developmental failure of the three pairs of primitive veins [6]. Although the reason for this remains unknown, several studies have suggested that it is caused by an intrauterine or perinatal thrombosis [7], while others have suggested embryonic dysontogenesis [4].

The diagnosis of KILT syndrome is mostly incidental. Patients most likely present to the hospital with unprovoked DVT. The clinical picture that should provoke the diagnosis of KILT syndrome is a young patient (second to fourth decade) with bilateral unprovoked DVT and a clear medical history [8].​​​​​​​​​​​​​​​​​​​​​ After confirming DVT by ultrasound and CT scan of the chest, abdomen, and pelvis, venography is recommended to confirm IVC and kidney abnormalities [9].

Currently, no clear management guidelines exist for KILT syndrome due to the rarity of the condition and a lack of studies. Because absent IVC can be a risk factor for DVT, the best treatment approach is vitamin K antagonists. However, because the duration of treatment is unknown, lifelong anticoagulant treatment and the use of elastic stockings are the gold standard treatments [10].

## Conclusions

The diagnosis of KILT syndrome is mostly incidental while investigating the complications associated with the syndrome. Currently, no clear guidelines exist to manage this syndrome due to the rarity of the condition. Management mostly relies on preventing possible complications such as pulmonary embolism and other thrombotic events.
